# Data-driven resuscitation training using pose estimation

**DOI:** 10.1186/s41077-023-00251-6

**Published:** 2023-04-16

**Authors:** Kerrin E. Weiss, Michaela Kolbe, Andrina Nef, Bastian Grande, Bravin Kalirajan, Mirko Meboldt, Quentin Lohmeyer

**Affiliations:** 1 Product Development Group Zurich, Department of Mechanical and Process Engineering, ETH Zurich, Leonhardstrasse 21, Zurich, 8092 Switzerland; 2 Simulation Center, University Hospital Zurich, Rämistrasse 100, 8091 Zurich, Switzerland; 3 Institute of Anaesthesiology, University Hospital Zurich, Rämistrasse 100, 8091 Zurich, Switzerland

**Keywords:** Education, Simulation, Feedback, Training, Pose estimation, Basic life support, Technology, Cardiopulmonary resuscitation, Assessment

## Abstract

**Background:**

Cardiopulmonary resuscitation (CPR) training improves CPR skills while heavily relying on feedback. The quality of feedback can vary between experts, indicating a need for data-driven feedback to support experts. The goal of this study was to investigate pose estimation, a motion detection technology, to assess individual and team CPR quality with the arm angle and chest-to-chest distance metrics.

**Methods:**

After mandatory basic life support training, 91 healthcare providers performed a simulated CPR scenario in teams. Their behaviour was simultaneously rated based on pose estimation and by experts. It was assessed if the arm was straight at the elbow, by calculating the mean arm angle, and how close the distance between the team members was during chest compressions, by calculating the chest-to-chest distance. Both pose estimation metrics were compared with the expert ratings.

**Results:**

The data-driven and expert-based ratings for the arm angle differed by 77.3%, and based on pose estimation, 13.2% of participants kept the arm straight. The chest-to-chest distance ratings by expert and by pose estimation differed by 20.7% and based on pose estimation 63.2% of participants were closer than 1 m to the team member performing compressions.

**Conclusions:**

Pose estimation-based metrics assessed learners’ arm angles in more detail and their chest-to-chest distance comparably to expert ratings. Pose estimation metrics can complement educators with additional objective detail and allow them to focus on other aspects of the simulated CPR training, increasing the training’s success and the participants’ CPR quality.

**Trial registration:**

Not applicable.

## Background

Cardiopulmonary resuscitation (CPR) quality is crucial for the outcome of cardiac arrests [1]. Resuscitation training programmes, such as basic and advanced cardiac life support, apply simulation as an educational tool [2–6]. Simulation-based education offers the possibility of rapid-cycle deliberate practice, mastery learning, and scripted debriefing, which are recommended strategies for improving resuscitation skills [2, 7]. These methods rely on assessment and feedback. Although expert feedback is certainly important [8, 9], the quality of the feedback provided can vary among educators and depends on their individual expertise [10]. Data-driven feedback and the ability to accurately assess CPR quality are required to mitigate the risks of inaccurate assessment, erroneous feedback, and negative learning [11–13]. Devices that provide automated and objective CPR feedback can improve the quality of both CPR and the subsequent debrief sessions [5, 14–19]. Because effective teamwork is vitally important during resuscitation [5, 20–22], the assessment of resuscitation skills should include team interaction aspects [23].

For this purpose, the use of motion detection technology has been investigated during simulation-based CPR training [24–28]. For example, Kinect-based motion sensing devices have been shown to improve chest compression quality [28] and detect mistakes during CPR training [29]. However, Kinect-based motion sensing devices require the placement of sensory markers on body parts to detect movements and calibrate instruments [28]. Disadvantages of the body marker technology include their potential for invading participants’ privacy, the time required for attaching markers and for performing the needed calibration, their interference with the natural behaviour of the participants, and their limited stability during CPR.

**Fig. 1:**
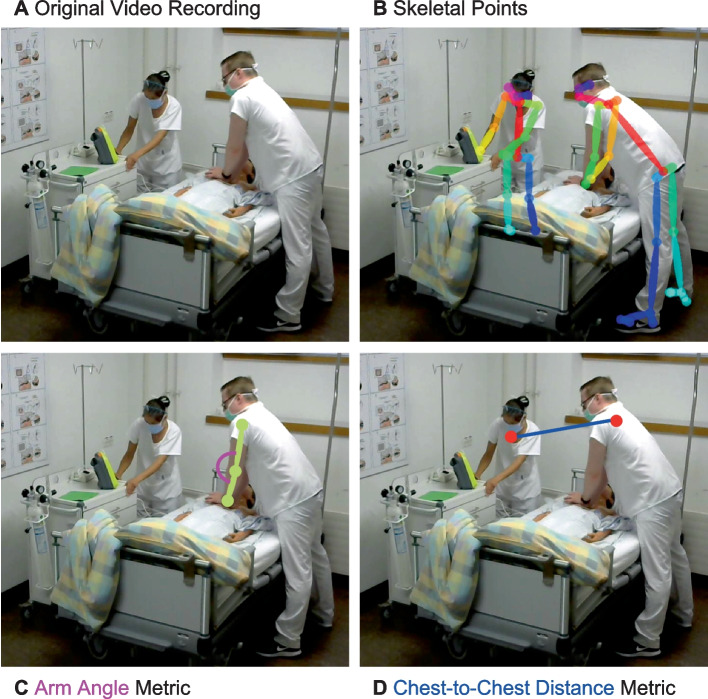
Pose estimation in CPR training **A** Original video recording of simulation-based CPR training **B** Pose estimation skeleton points (multiple colours) after calculation with the OpenPose [31] software **C** Arm angle (pink) pose estimation metric calculated for participant performing compressions **D** Chest-to-chest distance (blue) metric, calculated with the chest skeleton points of participants

In this study, we explore pose estimation [30, 31] as an alternative motion detection technology, which eliminates the need for marker placement. Pose estimation relies on real-time motion images, which are captured with two synchronized web cameras (Fig. 1A). Image analysis [32] enables the simultaneous detection of skeleton points and motion data of multiple study participants (Fig. 1B). Therefore, pose estimation may allow the assessment of CPR quality while tracking the interaction and motion range of team members during simulation-based training.

CPR quality is currently monitored using physiological measures, like invasive monitoring or end-tidal CO_2_, or quantitative measures, like feedback devices or visual assessment [33]. CPR feedback devices range from metronomes to audiovisual feedback. The most common are defibrillator-driven data and CPR “pucks” [17]. They improve the quality of chest compressions during training [17, 34, 35] and in real emergencies [36, 37]. Furthermore, the visual perception of CPR quality by healthcare providers is poor, showing the need for objective CPR quality metrics, either increasing the use of existing CPR feedback devices and their metrics or developing new methods to measure CPR quality objectively [33, 38].

This study aims to investigate the potential and feasibility of pose estimation in simulation-based CPR training with two first pose estimation metrics for supporting simulation training. These are the arm angle (Fig. 1C) to assess individual CPR quality and the chest-to-chest distance (Fig. 1D) to indicate team CPR quality. Importantly, these metrics are intended to inform and complement rather than substitute expert ratings of CPR quality using a data-driven approach.

## Methods

### Study participants

This study was approved by the Ethics Committee of Canton Zurich (BASEC number: Req-2020–00,200). All participants were healthcare providers at the University Hospital Zurich, Switzerland. They had signed up for statutory basic life support training. Upon arrival, training participants were invited to participate in the study. In total, 91 healthcare providers trained in 32 groups of two to four participants [39]. All included participants were able to consent, of legal age, and employed at the University Hospital Zürich as healthcare providers.

### Simulation-based CPR training and scenario

Participants attended the basic life support training as part of their mandatory training requirement. It included an online learning module and a practical training session in the simulation centre of the University Hospital Zurich led by simulation centre faculty. All course directors had a nursing background with special qualifications for either emergency, anaesthesia, or intensive care and were certified by the Swiss Resuscitation Council in basic life support (BLS) and automated external defibrillator (AED) use. The online learning module included the theory of BLS and AED use. It lasted between 30 and 60 min, and participants needed to pass a test to proceed with the 1-h practical training session. Practical training included the deliberate practice of chest compressions skills, ventilation skills, AED use skills, and lateral recumbent position on adult and child simulation manikins. It ended with participants performing a standardized resuscitation scenario with the following learning objectives based on the current BLS guidelines: call for help, organize workspace (i.e. make room and put the bed in a position optimal for CPR), leadership and communication among team members and with AED, chest compression in correct rhythm and position, rotating chest compressions every 2 min/when AED performs analysis, correct mask bag ventilation, and correct use of AED [11]. This scenario included a simulated patient (Ambu® Man, Ambu, Ballerup, Denmark) who was found unconscious in bed and was followed by a brief, structured debriefing.

### Data collection

Before data collection, the course director briefed the participants about the aim of the study and the overall recording set-up. Then, informed written consent was obtained. All participants invited agreed to participate and provided written consent. Two Logitech C270 webcams (Logitech, Lausanne, Switzerland), recording 30 frames per second, were used to record team behaviour after successful calibration via checkerboard.

### Metrics

Pose estimation was measured using the open-source software OpenPose [31], to detect human body skeleton points, e.g. chest, shoulder, hand, on single images. By applying OpenPose to the recorded videos, the participants’ 2D skeleton points were calculated. Then, the data was triangulated, using MATLAB (MathWorks, Natick, MA, USA), resulting in 3D skeletal points. Lastly, the metrics were calculated for all participants. The 2D arm angle was the first outcome of pose estimation, calculated for 53 participants performing chest compressions. Because effective CPR requires deep repetitive compression movements [40], the arms should be straight, that is, 180° rather than bent to avoid early fatigue, which is detrimental to performance [41–44]. In pose estimation, arms were considered straight if the mean arm angle was above 170° due to anatomical differences among participants and a standard deviation below 5° [43]. We chose to evaluate the arm angle using 2D not 3D data to investigate if a single camera is sufficient for drawing meaningful conclusions from pose estimation. CPR quality is often reported by using specific metrics of rate, depth/recoil, and leaning angle, aiming to measure the CPR quality. Pose estimation offers the opportunity to assess human posture, which impacts performance. Therefore, we suggest the additional quality metric of the arm angle.

The 3D chest-to-chest distance was the second calculated metric based on pose estimation, indicating the quality of team coordination [45–49]. It calculates the distance between the participant performing chest compression and each team member. The established CPR quality metrics and the arm angle focus on individual behaviour. Pose estimation provides the opportunity to include team metrics for exploration, as a first example, we choose the chest-to-chest distance between the caregivers. Movement and synchronicity are relevant measures for team coordination [50, 51], also in other team research domains, such as team sports, conceptual and empirical evidence can be found [48, 52]. In the context of resuscitation, the coordination of basic life support tasks [20, 53], information management, and CPR performance need to be coordinated simultaneously. Therefore, we explored chest-to-chest distance as a simple metric helping team members to coordinate. Within the context of the previously completed training, the teams were instructed to stay close to their team members for communication and task completion purposes (for example, positioning themselves and the defibrillator on the opposite of the patient’s bed from the team member performing chest compressions or in case the team switches roles). Standing too far from the person performing chest compressions over the simulations’ duration can indicate inactivity. Of the 87 chest-to-chest data sets, three could not be used due to superimposing of participants, which occurred if a participant stood directly behind another participant or object and was therefore not visible to the camera.

CPR quality was measured via expert rating, using the recorded videotapes of participants’ behaviour. Two experts, with 7 years and 20 years of CPR educator experience, independently rated arm angle and distance among team members as indicators of CPR quality resulting in one dichotomous score for the whole duration. They rated whether arms were straight during chest compressions [41, 42] and whether each participant was approximately 1 m away from the participant performing chest compressions [45–48]. To determine interrater reliability, all videos were coded separately by each expert.

### Statistical analysis

We used OpenPose [30] and MATLAB (MathWorks, Natick, MA, USA)—a programming language and numeric computing environment—to calculate 2D arm angle and 3D chest-to-chest distance based on pose estimation 2D skeleton points. We used SPSS Statistics 27 (IBM, Armonk, NY, USA) to calculate Cohen’s kappa for determining the interrater reliability among expert raters and for comparing expert ratings with computed pose estimation-based metrics [54].

## Results

The average age of study participants was 36.9 ± 10.2 years, and 53 participants (58.2%) were female. Their backgrounds included attending and resident physicians, medical students, physiotherapists, ergo therapists, midwives, and nurses. Their average professional experience was 10.4 ± 9.2 years, and 73 (80.2%) had already participated in basic life support training.

### Individual CPR quality: 2D arm angle

**Fig. 2:**
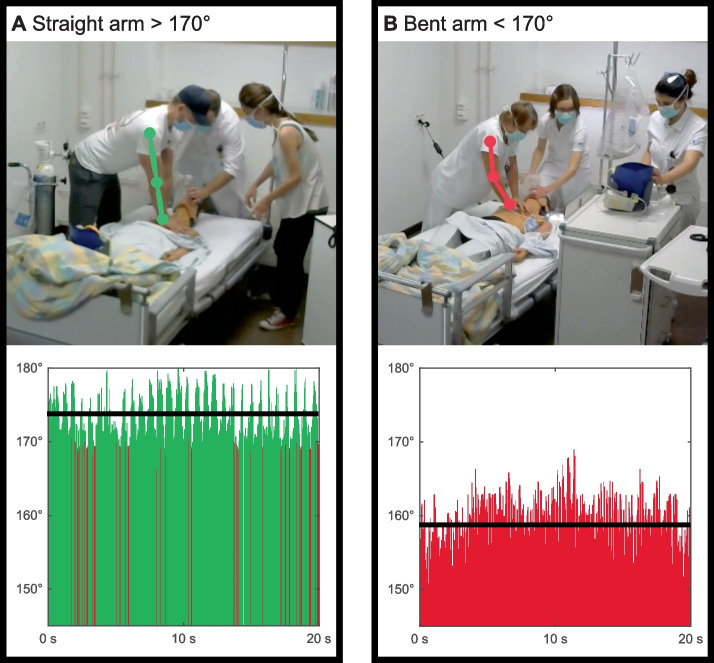
Arm angle **A** Exemplary straight arm angle skeletal points (green) visible in the video recording and participants’ arm angle with average (black) over the excerpt of 20 s (green: > 170°, red < 170°) **B** Exemplary bent arm angle skeletal points (red) visible in the video recording and participants’ arm angle with average (black) over 20 s (green: > 170°, red: < 170°)

Of the 91 study participants, 53 performed chest compressions. According to pose estimation calculations, the mean arm angle of all participants was 160.64 ± 8.39°. In total, 7 (13.2%) participants performed CPR with straight arms (Fig. 2A), and 46 (86.8%) participants performed CPR with bent arms (Fig. 2B). Expert ratings (*κ* = 0.88) showed opposing results: 48 (90.6%) participants performed CPR with straight arms, while 5 (9.4%) participants performed CPR with bent arms. The arm angle ratings based on pose estimation and based on experts differed for 77.3% (*κ* = 0.03) of the participants (Table 1).

**Table 1:** 2D arm angle measured with pose estimation. The mean arm angle of all participants performing chest compressions calculated with pose estimation, number of participants with straight arm during chest compressions calculated with pose estimation and rated by experts, and number of participants where pose estimation and expert ratings differed

**2D arm angle**	*n* = 53
Mean arm angle calculated with pose estimation	160.64 ± 8.39°
Participants with straight arm	
Calculated with pose estimation (> 170°)	7 (13.2%)
Rated by experts	48 (90.6%)
Difference pose estimation and expert ratings	41 (77.3%)

### Team CPR quality: 3D chest-to-chest distance

**Fig. 3:**
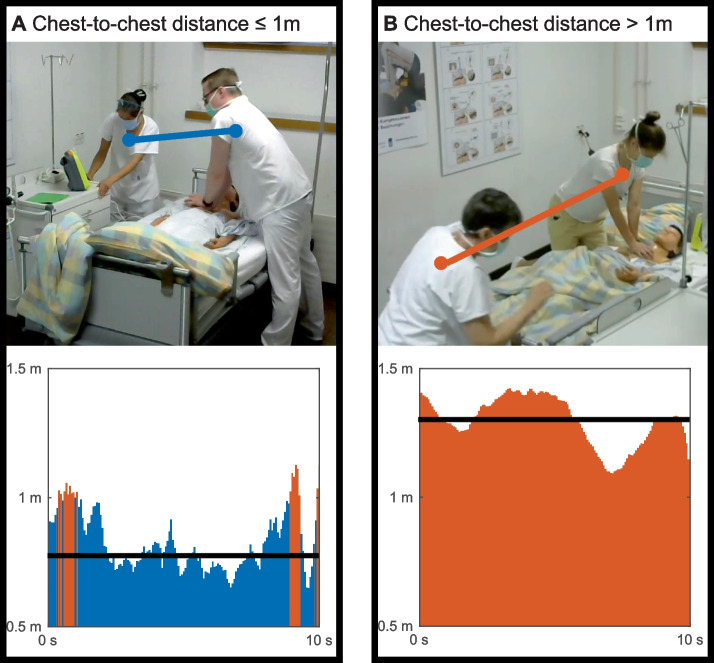
Chest-to-chest distance **A** Exemplary chest skeletal points and their close distance (blue) visible in the video recording and chest-to-chest distance with average (black) over 10 s (blue: ≤ 1 m, orange > 1 m) **B** Exemplary chest skeletal points and their far distance (orange) visible in the video recording and chest-to-chest distance with average (black) over the excerpt of 10 s (blue: ≤ 1 m, orange > 1 m)

The results for the chest distance were calculated between the participant performing chest compressions and the other team member, and in total, 87 distances were calculated. According to pose estimation calculations, the mean chest-to-chest distance of all participants combined was 1.03 ± 0.48 m. A distance of less than or exactly 1 m was obtained by 55 (63.2%) participants (Fig. 3A), and 32 (36.8%) were farther away than 1 m from the participant performing chest compressions (Fig. 3B). According to expert ratings (*κ* = 0.93), 51 (58.6%) participants stayed within close distance from each other whereas 36 (41.4%) did not. Pose estimation calculations of chest-to-chest distance differed in 20.7% (κ = 0.57) from expert ratings of chest-to-chest distance (Table 2).

**Table 2:** 3D chest-to-chest distance measured with pose estimation. The mean chest-to-chest distance of all calculated distances between team members and the participant performing chest compressions with pose estimation, number of participants with close chest-to-chest distance to participant performing chest compressions calculated with pose estimation and rated by experts, and number of participants where pose estimation and expert ratings differed

**3D chest-to-chest distance**	*n* = 87
Mean chest-to-chest distance calculated with pose estimation	1.03 ± 0.48 m
Participants with close chest-to-chest distance	
Calculated with pose estimation (≤ 1 m)	55 (63.2%)
Rated by experts	51 (58.6%)
Difference pose estimation and expert ratings	18 (20.7%)

## Discussion

We investigated pose estimation, a motion detection technology, to assess individual and team CPR quality with the arm angle and chest-to-chest distance metrics. For the arm angle metric, the expert and pose estimation-based ratings differed by 73.3%. We assume that this strong difference occurs because the cameras could capture the arm angle in more detail and are not prone to human perception error and bias [55, 56]. One may argue that the pose estimation was not overly strict. However, we defined the criterium for a straight arm at 170° rather than 180° to avoid an overly strict measure. If the arm angle metric were used as a single measure without expert context to assess individuals’ CPR competence, further investigation of criterium validity will be needed, especially since this detailed data is new and made possible using pose estimation. The use of technology to assess the arm angle additionally allows for the analysis of every training participant equally over the whole duration of the recording and may thus be a fruitful addition to traditional measures of CPR quality, especially in big group sizes [39]. The pose estimation-based evaluation of the arm angle suggests that only 13.2% had a straight arm during compressions, which strongly impacts compression depth [43]. Therefore, we recommend using this technology complementary to existing data-driven approaches during training to improve CPR quality, supporting instructors with objective data-driven metrics. The educators can integrate the specific knowledge provided by the metrics into the context of the whole scenario.

Concerning the chest-to-chest distance metric, both expert and pose estimation-based ratings, aligned and only differed by 20.7%. This shows that this pose estimation metric can be used to support experts and let them focus on other aspects during CPR training. The distance between the participants was less than or exactly 1 m in 63.2% of the cases. This indicator of team coordination might be strongly influenced by the COVID-19 situation at the time of data collection, when everyone was encouraged to physically distance themselves from others [57], although the minimum distance required cannot be adhered to in resuscitation scenarios. The chest-to-chest distance metric can be applied in different and new contexts to assess the distance between individuals. In CPR training, the chest-to-chest distance allows to point out individual deviations from the trained behaviour, allowing educators to focus on other aspects of the training.

Providing the average value for each metric for the whole simulation duration is the first step for pose estimation metrics, with the aim of demonstrating their potential. To increase their impact, the next step is to use the concept of “epochs” to analyse the pose estimation data [58, 59]. The results analysed in “epochs” and therefore reported in time intervals allow a more granular analysis of the recorded data, more informed feedback from instructors, and the learners to understand their behaviour in more detail and how it varied over the course of the whole simulation.

Our study has limitations. First, a limitation of pose estimation is that the data strongly depends on the camera position. Only what is visible can be used to calculate the skeleton points. This is especially relevant when metrics are calculated based on 2D data. Second, learners’ real behaviour might differ because of the simulated setting, but also because the data was collected directly after mandatory basic life support training. Third, the complementary use of pose estimation may not be limited to resuscitation training. We explored its feasibility only during simulated basic life support and consider its testing in other clinical and teamwork situations as one further next step.

Still, with the development of the two pose estimation-based metrics, we were able to provide quantitative and objective measures complementing the qualitative and subjective knowledge of experts with extensive experience. This data-driven approach can free up the cognitive capacity of instructors so that they can focus on other aspects of the training scenario [60]. The metrics provide objective feedback, which improves BLS skills significantly [16]. We envision a combination of various approaches, e.g. defibrillator-driven data, CPR “puck”, and pose estimation creating a versatile and holistic approach to measure CPR quality and to provide data-driven feedback to training participants [61]. Pose estimation metrics complement the data from defibrillators and “pucks”, e.g. compression rate, depth, and effectiveness, with information on the body posture, arm position, and body position for one or multiple people.

Although the development of pose estimation metrics is complex and time-consuming, once they are implemented, they take on low effort and cost. The two exemplary metrics described in this study are representative of all the possible metrics that can be calculated based on pose estimation. Metric-based measurements are easily reproducible and allow for quick comparisons to behaviours from former training sessions, which would allow the observation of CPR skill development. The fact that no body markers are needed for pose estimation can support the immersion during simulation-based training and therefore can increase the transfer from training to real emergency situations. Furthermore, pose estimation metrics can be used additionally to CPR feedback/prompt devices during training which improves CPR skill acquisition and retention [17]. To determine if pose estimation metrics improve patient outcomes, further studies are needed. Possible future applications include measuring training success, transferring the demonstrated metrics to other scenarios, or developing new technology-based metrics.

## Conclusion

In this first and exemplary study, we investigated the potential and feasibility of pose estimation metrics to support resuscitation educators’ simulation-based training. The individual arm angle metric allowed for a more detailed and objective assessment of CPR quality while the team chest-to-chest distance assessed participants’ behaviour equally compared to expert ratings. Thus, data-driven metrics can support educators, by providing complementary feedback data and allowing them to focus on other aspects of their learners’ resuscitation skills during training or supporting them with additional details, therefore increasing the training quality and participants’ CPR quality.

## Data Availability

The data sets used during the current study are available from the corresponding author upon reasonable request.

## Abbreviations

AED: Automated external defibrillator
BLS: Basic life support
CPR: Cardiopulmonary resuscitation
